# The effects of dietary saturated fat source on weight gain and adiposity are influenced by both sex and total dietary lipid intake in zebrafish

**DOI:** 10.1371/journal.pone.0257914

**Published:** 2021-10-22

**Authors:** Lauren A. Fowler, Audrey D. Powers, Michael B. Williams, James L. Davis, Robert J. Barry, Louis R. D’Abramo, Stephen A. Watts

**Affiliations:** 1 Department of Biology, University of Alabama at Birmingham, Birmingham, Alabama, United States of America; 2 Nutrition Obesity Research Center, University of Alabama at Birmingham, Birmingham, Alabama, United States of America; 3 University of Alabama at Birmingham, Birmingham, Alabama, United States of America; University of Life Sciences in Lublin, POLAND

## Abstract

The effects of saturated fat intake on obesity and cardiovascular health remain inconclusive, likely due in part to their varied nature and interactions with other nutrients. Investigating the synergistic effects of different saturated fat sources with other dietary lipid components will help establish more accurate nutritional guidelines for dietary fat intake. Over the past two decades, zebrafish (*Danio rerio*) have been established as an attractive model system to address questions regarding contributions of dietary lipid intake to diet-induced obesity in humans. The goal of the present study was to assess interactions of three different saturated fat sources (milk fat, palm oil, and coconut oil) with sex and total dietary lipid intake on weight gain and body composition in adult zebrafish. Larvae were raised on live feeds until 28 days post fertilization, and then fed a formulated maintenance diet until three months of age. An eight-week feeding trial was then initiated, in which zebrafish were fed nine experimental low- and high-fat diets varying in saturated fatty acid and long-chain polyunsaturated fatty acid content, in addition to a low-fat and high-fat control diet. At termination of the feeding trial, each treatment was evaluated according to body mass, moisture content, and adiposity. Sex and diet significantly interacted in their effects on body mass (*P* = 0.026), moisture content (*P* = 0.044), and adiposity (*P* = 0.035). The influence of saturated fat source on body mass was observed to be dependent on intake of total dietary lipid. In females, all three saturated fat sources had similar effects on adiposity. From these observations, we hypothesize that impacts of saturated fat intake on energy allocation and obesity-related phenotypes are influenced by both sex and intake of other dietary lipid components. Our results suggest that current nutritional guidelines for saturated fat intake may need to be re-evaluated and take sex-specific recommendations into consideration.

## Introduction

Characterizing the effects of saturated fat intake on obesity and metabolic health continues to be a major challenge. In previous decades, dietary guidelines have promoted reducing the intake of total dietary fat and sources of saturated fatty acids (SFA), and more recently, replacing SFAs with sources of long-chain polyunsaturated fatty acids (LC-PUFA) [1]. However, multiple reviews and meta-analyses conducted over the last decade have revealed inconclusive evidence regarding the actual effects of saturated fat intake on obesity and cardiovascular health [1–4]. Individual SFAs vary widely in their physiological effects on health; therefore, it is important to consider the specific dietary source of SFAs consumed [3, 5]. For example, many studies using humans and animals have reported that consumption of medium chain triglycerides (MCTs) has resulted in reductions in body weight and adiposity and may even confer beneficial effects on cardiovascular disease risk [6, 7].

Another factor that should be carefully considered is the balance of SFAs with other dietary lipid components [1]. Previous studies have noted that diverse effects of high fat diets on metabolic health may be attributed to variations in fatty acid profiles, which suggests that fat quality may be as important as fat quantity [8, 9]. Therefore, a better understanding is needed regarding an optimal balance of dietary lipid. Additionally, sexually dimorphic responses to dietary lipid manipulation should also be carefully considered in these studies [10].

To address these identified gaps in knowledge, many researchers have evaluated animal models to answer these questions. Similar to humans, zebrafish exhibit increases in weight gain, adiposity, and metabolic disease risk in response to a high-fat diet [11–13]. Furthermore, zebrafish also exhibit different patterns of digestion, transportation, and metabolism in response to short-, medium-, and long-chain fatty acids [14].

In the current study, we aimed to investigate the interactions of various dietary lipid components with three different saturated fat sources (coconut oil, palm oil, and milk fat) on weight gain and body composition in male and female adult zebrafish. Specifically, we wanted to test two main hypotheses: 1) that different combinations of dietary saturated fat sources, LC-PUFA content, and total intake of dietary fat can interact in their effects on obesity and 2) that sex interacts with the influence of dietary lipid composition on body mass and adiposity. We describe interactive effects of total dietary lipid intake, saturated fat source, and sex on obesity-related phenotypes in zebrafish.

## Materials and methods

### Diet preparation

**Fig 1:**
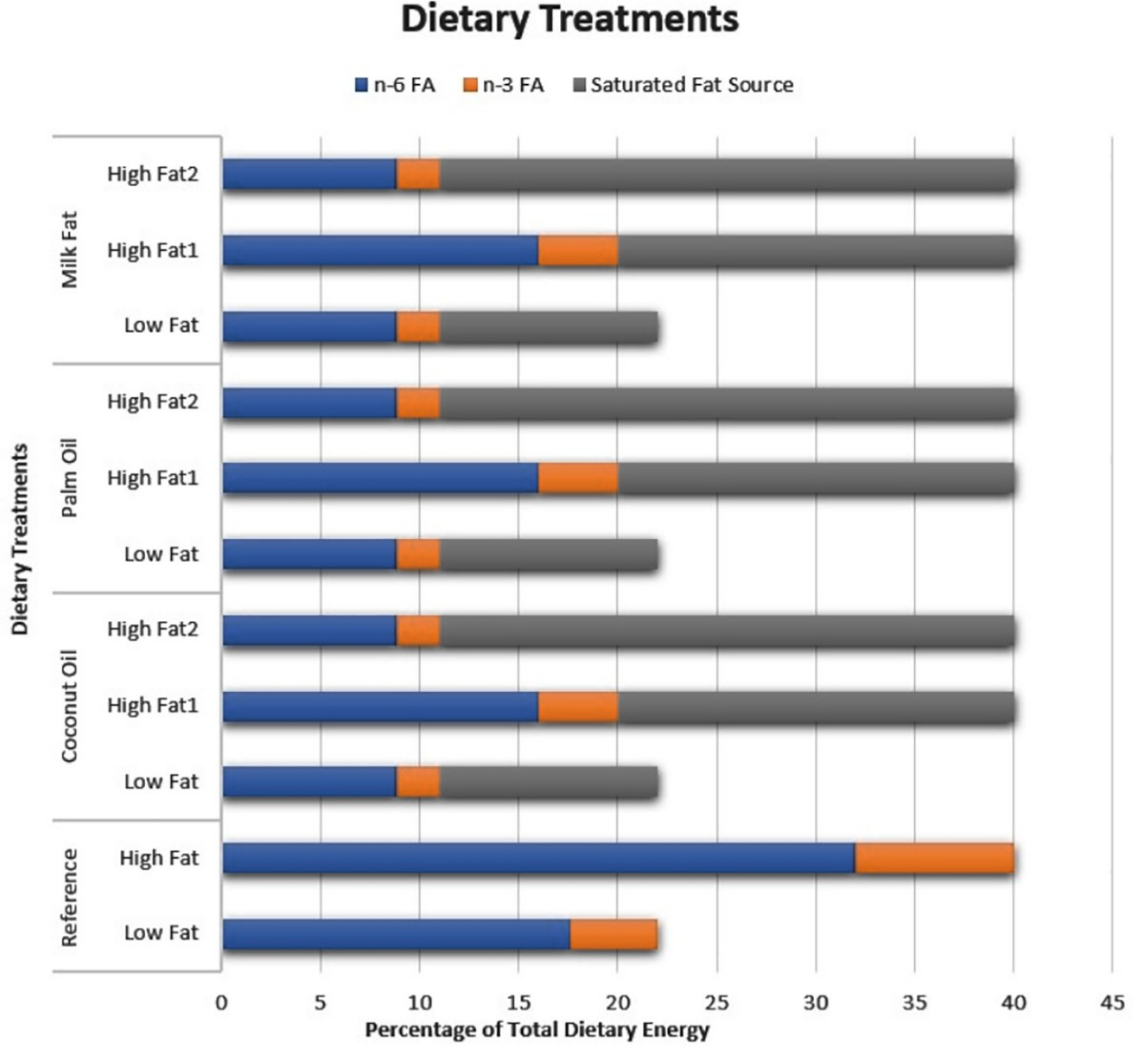
Comparison of dietary treatments by energy content and saturated fat source.

Eleven chemically defined diets were formulated and contained purified and semi-purified ingredients (Table 1, Fig 1). The two primary sources of LC-PUFAs included in all diets were safflower seed oil (food grade,Sigma-Aldrich, Inc.) and menhaden fish oil (Virginia Prime^®^ Gold Fish Oil, Omega Protein Inc, Houston, TX). Safflower seed oil was used as the principal source of n-6 LC-PUFA, while menhaden fish oil was used as the principal source of n-3 LC-PUFA. All diets contained a 4:1 ratio of safflower oil to menhaden oil. The sources of saturated fat used in the experimental diets were coconut oil (Sigma-Aldrich, Inc), palm fruit oil (Sigma-Aldrich, Inc), and anhydrous milk fat (Envigo Teklad Diets). The estimated fatty acid content of all primary lipid sources can be found in Table 2. In all diets, levels of total dietary fat were adjusted with Alpha-Cel^TM^, a non-nutritive bulking agent (MP Biomedicals, LLC, Solon, OH). The carbohydrate and protein content remained constant among all eleven dietary treatments.

**Table 1:** Composition of reference and experimental diets (as fed).

	Reference diets		Experimental diets		
	Low-fat	High-fat	Low-fat	High-fat 1	High-fat 2
**Ingredient (g/100 g)**					
Saturated fat source^a^	-	-	1.50	8.10	14.63
Safflower oil^b^	2.10	10.80	1.05	5.40	1.05
Menhaden fish oil^c^	1.05	5.40	0.52	2.70	0.52
Alpha cellulose	16.04	2.99	18.96	2.99	2.99
Casein-vita free	25.00	25.00	25.00	25.00	25.00
Fish protein hydrosylate	25.00	25.00	25.00	25.00	25.00
Soy protein isolate	6.60	6.60	6.60	6.60	6.60
Wheat starch	9.34	9.34	9.34	9.34	9.34
Dextrin	2.16	2.16	2.16	2.16	2.16
Alginate	3.00	3.00	3.00	3.00	3.00
Soy lecithin (refined)	3.00	3.00	3.00	3.00	3.00
Vitamin mix^d^	2.00	2.00	2.00	2.00	2.00
Mineral mix BTm^e^	3.00	3.00	3.00	3.00	3.00
Canthaxanthin (10%)	1.00	1.00	1.00	1.00	1.00
K phosphate monobasic	1.15	1.15	1.15	1.15	1.15
Glucosamine	0.25	0.25	0.25	0.25	0.25
Betaine	0.15	0.15	0.15	0.15	0.15
Cholesterol	0.12	0.12	0.12	0.12	0.12
Ascorbylpalmitate	0.04	0.04	0.04	0.04	0.04
**Nutrient content (calculated)**					
Lipid (%)	9.09	22.14	9.09	22.14	22.14
Protein (%)	49.82	49.82	49.82	49.82	49.82
Carbohydrate (%)	12.16	12.16	12.16	12.16	12.16
Energy (cal/g)	4160	5393	4160	5393	5393

^a^ Palm fruit oil, coconut oil, or anhydrous milk fat.

^b^Sigma-Aldrich, Cat no. S8281.

^c^Virginia Prime Gold, Omega Protein.

^d^Composition of MP Vitamin Diet Fortification Mixture (%): p-aminobenzoic acid, 0.500; ascorbic acid, 4.500; biotin, 0.002; calcium pantothenate, 0.300; choline chloride, 7.500; DL-α-tocopherol acetate, 2.200; folic acid, 0.009; inositol, 0.5; menadione, 0.225; niacin, 0.425; pyridoxine hydrochloride, 0.100; riboflavin, 0.100; thiamine hydrochloride, 0.100; vitamin A acetate (500,000 IU/gm), 0.180; vitamin B_12_, 0.000135; vitamin D_2_ (850,000 IU/gm), 0.0125.

^e^Composition of the mineral premix (%): calcium carbonate, 2.100; calcium phosphate dibasic, 73.500; citric acid, 0.227; cupric citrate, 0.046; ferric citrate, 0.558; magnesium oxide, 2.500; magnesium citrate, 0.835; potassium iodide, 0.001; potassium phosphate dibasic, 8.100; potassium sulfate, 6.800; sodium chloride, 3.060; sodium phosphate, 2.140; zinc citrate, 0.133.

**Table 2:** Estimated fatty acid content of primary lipid sources.

Fatty acid content	SAF^a^	MFO^b^	CO^a^	PFO^a^	AMF^c^
**Saturated fatty acids**					
C08:0 Octanoic (Caprylic)	-	-	8.0%	-	1.0%
C10:0 Decanoic (Capric)	-	-	6.4%	-	2.0%
C12:0 Dodecanoic (Lauric)	-	-	48.5%	0.2%	3.1%
C14:0 Tetradecanoic (Myristic)	0.1%	8.04%	18.0%	1.1%	11.7%
C16:0 Hexadecanoic (Palmitic)	6.0–7.5%	16.85%	8.0%	44.0%	26.2%
C18:0 Octadecanoic (Stearic)	2.0–2.5%	3.09%	2.5%	4.5%	12.5%
C20:0 Eicosanoic (Arachidic)	0.5%	-	-	0.1%	-
C22:0 Docosanoic	-	-	-	-	-
C24:0 Tetracosanoic	-	-	-	-	-
Σ Saturated fatty acid content	9%	29%	91.6%	49.8%	65.0%
**Monounsaturated fatty acids**					
C14:1 Tetradecenoic (Myristoleic)	-	-	-	-	-
C16:1 Hexadecenoic (Palmitoleic)	0.1%	11.50%	-	-	1.9%
C18:1n-9 Octadecenoic (Oleic)	12.0%	9.74%	6.5%	39.2%	28.2%
C20:1n-9 Eicosenoic (Gadoleic)	0.3%	-	-	-	-
C22:1n-9 Docosenoic (Erucic)	-	-	-	-	-
C24:1n-9 Tetracosenoic (Nervonic)	-	-	-	-	-
Σ Monounsaturated fatty acid content	13%	21.3%	6.5%	40%	31.0%
**N-6 polyunsaturated fatty acids**					
C18:2n-6 Octadecadienoic (Linoleic)	70.0%	1.89%	1.5%	10.1%	2.9%
C18:3n-6 Octadecatrienoic (GLA)	-	-	-	-	-
C20:2n-6 Eicosadienoic	-	-	-	-	-
C20:3n-6 Eicosatrienoic (DGLA)	-	-	-	-	-
C20:4n-6 Eicosatetraenoic (Arachidonic)	-	-	-	-	-
C22:2n-6 Docosadienoic	-	-	-	-	-
C22:4n-6 Docosatetraenoic (Adrenic)	-	-	-	-	-
C22:5n-6 Docosapentaenoic (Osbond)	-	-	-	-	-
Σ n-6 fatty acid content	72.0%	5.30%	1.8%	10.3%	3.0%
**N-3 polyunsaturated fatty acids**					
C18:3n-3 Octadecatrienoic (α- Linolenic)	3.3%	2.20%	-	0.4%	0.5%
C18:4n-3 Octadecatetranoic (Stearidonic)	-	3.21%	-	-	-
C20:3n-3 Eicosatrienoic	-	-	-	-	-
C20:4n-3 Eicosatetraenoic	-	2.49%	-	-	-
C20:5n-3 Eicosapentaenoic (EPA)	-	14.05%	-	-	-
C21:5n-3 Heneicosapentaeonic	-	-	-	-	-
C22:3n-3 Docosatrienoic	-	-	-	-	-
C22:5n-3 Docosapentaenoic	-	2.95%	-	-	-
C22:6n-3 Docosahexaenoic (DHA)	-	12.26%	-	-	-
Σ n-3 fatty acid content	3.6%	36.65%	<1%	0.4%	0.5%

Abbreviations: AMF, anhydrous milk fat; CO, coconut oil; MFO, menhaden fish oil; PFO, palm fruit oil; SAF, safflower oil.

^a^Sigma-Aldrich, Cat nos. S8281 (safflower oil), C1758 (coconut oil), and W530216 (palm fruit oil).

^b^Virginia Prime Gold, Omega Protein.

^c^Envigo Teklad Diets, Product Code CA.0366.

For the reference diets, the total amount of dietary lipid was adjusted only with the safflower and fish oils and did not include any of the three sources of saturated fat. For the other diets, the amounts of each dietary lipid source (LC-PUFA and saturated fat) were adjusted to achieve both a desired total quantity (low-fat vs. high-fat) and desired composition of lipid for each diet. For each source of saturated fat, a low-fat diet and two high-fat diets (high-fat 1 and high-fat 2) were prepared. Compared to the low-fat diet, the high-fat 1 diet had the same ratio of saturated fat to LC-PUFA sources, but different amounts of each LC-PUFA source added. Relative to the high-fat 1 diet, the high-fat 2 diet had a higher ratio of saturated fat to LC-PUFA source but contained the same amount of both LC-PUFA sources as the low-fat diet.

Feed ingredients were weighed using a Mettler Toledo analytical balance. All diets were formulated with a single, common base mix (excluding the lipid sources and alpha-cellulose). The ingredients for the base mix were combined first using a Kitchen Aid Professional 600.

Orbital Mixer. Alpha-cellulose was then measured out and mixed in after dividing the base mix for the low- and high-fat diets. The safflower and menhaden oils were then weighed and added to each diet using a Cuisinart Food Processor. Finally, the saturated fat sources were weighed and added to the experimental diets. To ensure that the saturated fat sources were evenly incorporated, all solid sources were melted in hot water prior to mixing. Diets were then extruded with a Kitchen Aid Extruder (KPEXTA) fitted with a pasta maker attachment. Feed strands were air-dried on wire trays for 24 hours, and then stored in storage bags at 4˚C until used. Feed was ground to a powder (250–500 μm sieved) prior to feeding.

### Experimental protocols

This study was conducted using recommendations of the Guide for the Care and Use of Laboratory Animals, National Resource Council. All procedures abided by standard zebrafish husbandry requirements for housing and euthanasia, and all efforts were made to minimize suffering. Protocols were reviewed and approved by the Institutional Animal Care and Use Committee (IACUC) at the University of Alabama at Birmingham.

Throughout the course of the experiment, zebrafish were maintained at a water temperature of 28°C and 1500 μS/cm conductivity in re-circulating systems (Aquaneering, Inc.) with mechanical, chemical, and biological filtration, along with UV sterilization. Synthetic sea salts (Instant Ocean) were added to maintain conductivity at 1500 μS/cm, and if required, sodium bicarbonate was added to sustain the pH of the system water at ~7.4. Before being added to the Aquaneering systems, water was conditioned with filtration through a 5 μm sediment filter, charcoal filter, reverse osmosis system, and a cation/anion exchange resin (Kent Marine). At minimum, 20% of water from each system was exchanged on a weekly basis. Within each tank, flow rates were adjusted to provide a minimum of two water changes each hour. Tanks were siphoned weekly to remove any uneaten food or debris. To ensure that acceptable water quality standards were sustained, total ammonia nitrogen, nitrite, and nitrate were monitored weekly with colorimetric tests (Mars Fish Care, Inc.). A 14-hour light/10-hour dark cycle was sustained throughout the duration of the experiment.

Zebrafish embryos (wild-type, AB strain) were first obtained from the Nutrition Obesity Research Center’s Aquatic Animal Resource Core at UAB, where they were collected from a mass spawn of adult zebrafish. Embryos were subsequently transferred to Petri dishes (n = 75 embryos per dish) and incubated at 28.5°C until five days post fertilization (dpf). Feeding protocols for the experiment were then divided into three phases. Due to the large number of animals required for this experiment, zebrafish were divided into two cohorts and stocked 3 months apart. The experimental protocols described were strictly followed for both cohorts.

#### Phase I

When fish reached 5 dpf, Phase I of the feeding protocol was initiated. From 5–10 dpf, hatched larvae were polycultured with the rotifer *Branchionus plicatilis* at a salinity of 5 ppt and enriched with 5 mL of *Nannochloropsis* (RotiGrow Omega, Reed Mariculture). While in polyculture, larvae were maintained in 24 static 6-liter tanks (48 tanks total for both cohorts combined) at a density of 75 larvae per tank. At 11 dpf, each 6-liter tank was placed on a slow drip and fed 10 mL of stage I *Artemia nauplii* (>300 nauplii per fish) twice daily until fish reached 28 dpf.

#### Phase II

At 28 dpf, Phase II of the experiment (the maintenance/grow-out period) was initiated. Fish from all 6-liter tanks were then re-distributed into 20 6-liter tanks (40 tanks total for both cohorts combined) at a density of 70 fish per tank. All animals in the study were then fed a single chemically defined, formulated maintenance diet (Table 3) until three months of age. Throughout Phase II, fish in each tank received a daily ration equal to 4–5% of body mass. To maintain this ration, the group weights for each tank were recorded on a bi-weekly basis. The daily ration was then determined for each tank and adjusted accordingly.

**Table 3:** Composition of maintenance (grow-out) diet.

Ingredient	Amount (g/100 g)
Safflower oil^a^	2.33
Menhaden fish oil^b^	4.67
Alpha cellulose	1.00
Casein-vita free	25.00
Fish protein hydrosylate	20.00
Soy protein isolate	5.00
Wheat gluten	7.00
Wheat starch	9.60
Dextrin	5.00
Alginate (TIC algin 400)	5.38
Soy lecithin (refined)	4.00
Vitamin mix^c^	4.00
Mineral mix BTm^d^	3.00
Canthaxanthin (10%)	2.31
Potassium phosphate monobasic	1.15
Glucosamine	0.25
Betaine	0.15
Cholesterol	0.12
Ascorbylpalmitate	0.04

^a^Sigma-Aldrich, Cat no. S8281.

^b^Virginia Prime Gold, Omega Protein.

^c^Composition of MP Biomedicals Vitamin Diet Fortification Mixture (%): p-aminobenzoic acid, 0.500; ascorbic acid, 4.500; biotin, 0.002; calcium pantothenate, 0.300; choline chloride, 7.500; DL-α-tocopherol acetate, 2.200; folic acid, 0.009; inositol, 0.5; menadione, 0.225; niacin, 0.425; pyridoxine hydrochloride, 0.100; riboflavin, 0.100; thiamine hydrochloride, 0.100; vitamin A acetate (500,000 IU/gm), 0.180; vitamin B_12_, 0.000135; vitamin D_2_ (850,000 IU/gm), 0.0125.

^d^Composition of the mineral premix (%): calcium carbonate, 2.100; calcium phosphate dibasic, 73.500; citric acid, 0.227; cupric citrate, 0.046; ferric citrate, 0.558; magnesium oxide, 2.500; magnesium citrate, 0.835; potassium iodide, 0.001; potassium phosphate dibasic, 8.100; potassium sulfate, 6.800; sodium chloride, 3.060; sodium phosphate, 2.140; zinc citrate, 0.133.

#### Phase III

After fish reached three months of age, Phase III (the experimental feeding trial) was initiated. At this time, fish from all 6-liter tanks were combined and sexed. Fish were then randomly distributed into 2.8-liter tanks (77 tanks per cohort, 154 tanks total) at a density of 14 fish per tank (7 males and 7 females). After stocking, tanks were randomly assigned to one of 11 treatments (n = 14 tanks total per treatment and 7 tanks within each cohort) and randomly distributed over two Aquaneering rack systems (n = 38–39 tanks per system). After placement on the systems, fish were fed experimental diets for an eight-week period. Prior to stocking and initiation of the eight-week feeding trial, a sub-sample of 48 fish (24 males and 24 females) was randomly selected from each cohort to obtain initial measures of body mass. These measures were used to estimate the starting body mass for all treatments within each cohort.

During the experimental feeding trial, fish in each tank were fed a daily ration of approximately 7% of body mass. Similar to Phase II, wet weights from each tank of fish were recorded on a bi-weekly basis during Phase III to maintain this 7% ration. For each weigh period, fish were quickly weighed as a group using a tared scale and subsequently returned to the re-circulating system. Using the calculated averages of body mass from each tank within a diet, the daily ration was then adjusted accordingly for each dietary treatment.

### Euthanasia

Fish were euthanized by rapid submersion in ice water for a minimum of ten minutes after cessation of all opercular movement was observed.

### Experiment termination

At the termination of the feeding trial, each fish in the study was assigned a unique identification number for which sex, terminal body mass, length, and randomly assigned outcome were recorded. Fish were first euthanized, and then weighed individually on a tared scale and photographed from above. The wet body mass of each fish was recorded to 0.001 g. After weights and photographs were recorded, individuals randomized to body composition analysis were stored at -20°C until analysis. Sample sizes for all outcomes of interest can be found in Table 4.

**Table 4:** Sample sizes by treatment and sex.

	Body mass		Moisture content		Total body lipid	
	M	F	M	F	M	F
**Reference diets**						
Low**-f**at	95	91	24	24	24	24
High**-f**at	83	96	24	23	24	24
**Coconut oil diets**						
Low**-f**at	93	90	25	26	24	24
High**-f**at 1	102	90	24	25	24	24
High**-f**at 2	101	89	24	25	23	24
**Palm fruit oil diets**						
Low**-f**at	87	105	25	25	23	23
High**-f**at 1	93	97	24	23	24	23
High**-f**at 2	91	98	24	26	24	24
**Anhydrous milk fat diets**						
Low**-f**at	84	107	24	24	24	24
High**-f**at 1	88	89	25	25	24	24
High**-f**at 2	92	92	26	25	24	24

### Body composition parameters

From each dietary treatment, 24 males and 24 females were reserved for assessment of body composition. All weights for body composition measurements were recorded to 0.0001 g. Carcasses were removed from storage at -20°C, thawed, weighed, placed in individual aluminum pans, and dried in a 50°C oven for 120 hours. After removal from the oven, samples were re-weighed to determine dry body mass. Body water mass was then calculated for each sample by subtracting dry body mass from wet body mass. Moisture content (percent body water) was determined for each carcass with the following formula:

$$\mathrm{Moisture}\;\mathrm{content}=\left(\frac{\mathrm{Body}\;\mathrm{water}\;\mathrm{mass}\;(\mathrm{mg})}{\mathrm{Wet}\;\mathrm{body}\;\mathrm{mass}\;(\mathrm{mg})}\right)\;X\;100$$

Adiposity was assessed with chemical carcass analysis. Briefly, total lipid mass was gravimetrically determined from each dried carcass by extraction with chloroform and methanol. The protocol for total lipid extraction was a modified version of the Folch method [15] and has been described in detail elsewhere [16]. Adiposity (lipid content) was calculated with the following formula:

$$\mathrm{Lipid}\;\mathrm{content}=\left(\frac{\mathrm{Total}\;\mathrm{lipid}\;\mathrm{mass}\;x\;1.25}{\mathrm{Dry}\;\mathrm{body}\;\mathrm{mass}}\right)\;X\;100$$

### Statistical modeling and analysis

All data are presented as means ± standard error. Analyses and figures were generated with R Statistical Software (R Core Team, 2016, v3.4.2). Figures were produced with help of the “ggplot2” and “ggpubr” packages [17, 18]. Analyses for all outcomes of interest were stratified by males and females. *P* < 0.05 was considered statistically significant for all outcomes.

With the “lme4” and “lmerTest” packages in R [19, 20], we used a mixed effects model approach to evaluate differences among outcomes for wet body mass, moisture content, and adiposity/total lipid mass. The mixed models were fitted by restricted maximum likelihood (REML) and used applied Satterthwaite approximation for degrees of freedom. Diet, cohort, and sex were evaluated as fixed effects, while tank was included as a random effect in all models to control for any potential environmental conditions that varied among tanks. Interactions of diet with sex and cohort were also tested for each outcome. Body composition samples were analyzed over a three-month period, with 48 samples processed per week (per batch). To control for any unknown variability attributed to batch, models for moisture content and adiposity also controlled for batch as a random effect. For analyses where diet was significantly associated with the outcome of interest, a planned comparison of means was conducted for five specific diet groups (Table 5) with help of the “multcomp” package [21]. All planned comparisons controlled for an inflated Type I error rate using the Tukey’s Honest Significant Difference method.

**Table 5:** Diet comparison groups for planned pairwise comparisons.

Comparison group	Diets included
One	All low-fat diets (Reference, coconut, palm, and milk fat)
Two	High-fat reference and high-fat 2 diets (coconut, palm, and milk fat)
Three	Coconut oil diets (low-fat, high-fat 1, and high-fat 2)
Four	Palm oil diets (low-fat, high-fat 1, and high-fat 2)
Five	Milk fat diets (low-fat, high-fat 1, and high-fat 2)

To ensure all assumptions were met, data were first assessed for normality and equal variances. Any outcome or predictor variables not meeting the assumption of normality were log-transformed prior to analysis. Outcomes analyzed as percentages were also log-transformed prior to analysis. Model fit was then confirmed with a normal distribution of residuals and residual plots were examined to ensure homoscedasticity. Model assumptions were confirmed with use of the “car” package [22].

## Results

Survivorship surpassed 95% in all dietary treatments. All diets promoted growth and weight gain over the course of the study, with no apparent limitations in palatability observed. Contributions of tank and batch to data variability were found to be minimal in all analyses. Significant interactions of cohort and diet were not observed in any of the outcomes evaluated.

### Wet body mass

Overall, females were larger than males, with mean wet body mass (WBM) among all diets ranging from 352 ± 6.3 mg to 394 ± 6.4 mg in males, and in females, from 468 ± 21 mg to 617 ± 26 mg. Both diet and sex were observed to have a significant main effect on WBM (Table 6). Cohort was also independently associated with WBM. For reasons that were unclear, fish from cohort 1 gained more body mass compared to fish from cohort 2 (mean WBM, 545 ± 6.0 mg vs. 373 ± 3.3 mg, respectively). Despite this difference in magnitude, both cohorts exhibited similar trends in mean WBM among diets.

**Table 6:** Main and interactive effects of diet, sex, and cohort on wet body mass^a^.

Model^b^	Fixed effect	Sum of squares	df	Mean squares	F-statistic	Pr(>F)
**All**	Diet	2.468	10	0.247	4.680	<0.001
	Sex	40.320	1	40.320	764.209	<0.001
	Cohort	22.950	1	22.950	435.040	<0.001
	Diet*sex	1.078	10	0.108	2.040	0.026
**Males**	Diet	0.707	10	0.071	3.742	<0.001
	Cohort	3.955	1	3.955	209.164	<0.001
	Diet*cohort	0.410	10	0.746	0.746	0.680
**Females**	Diet	2.460	10	0.246	3.660	<0.001
	Cohort	24.448	1	24.448	363.580	<0.001
	Diet*cohort	1.286	10	0.129	1.910	0.051

^a^Log-transformed for analysis.

^b^Analyzed with mixed effects models, which controlled for tank as a random effect.

**Fig 2:**
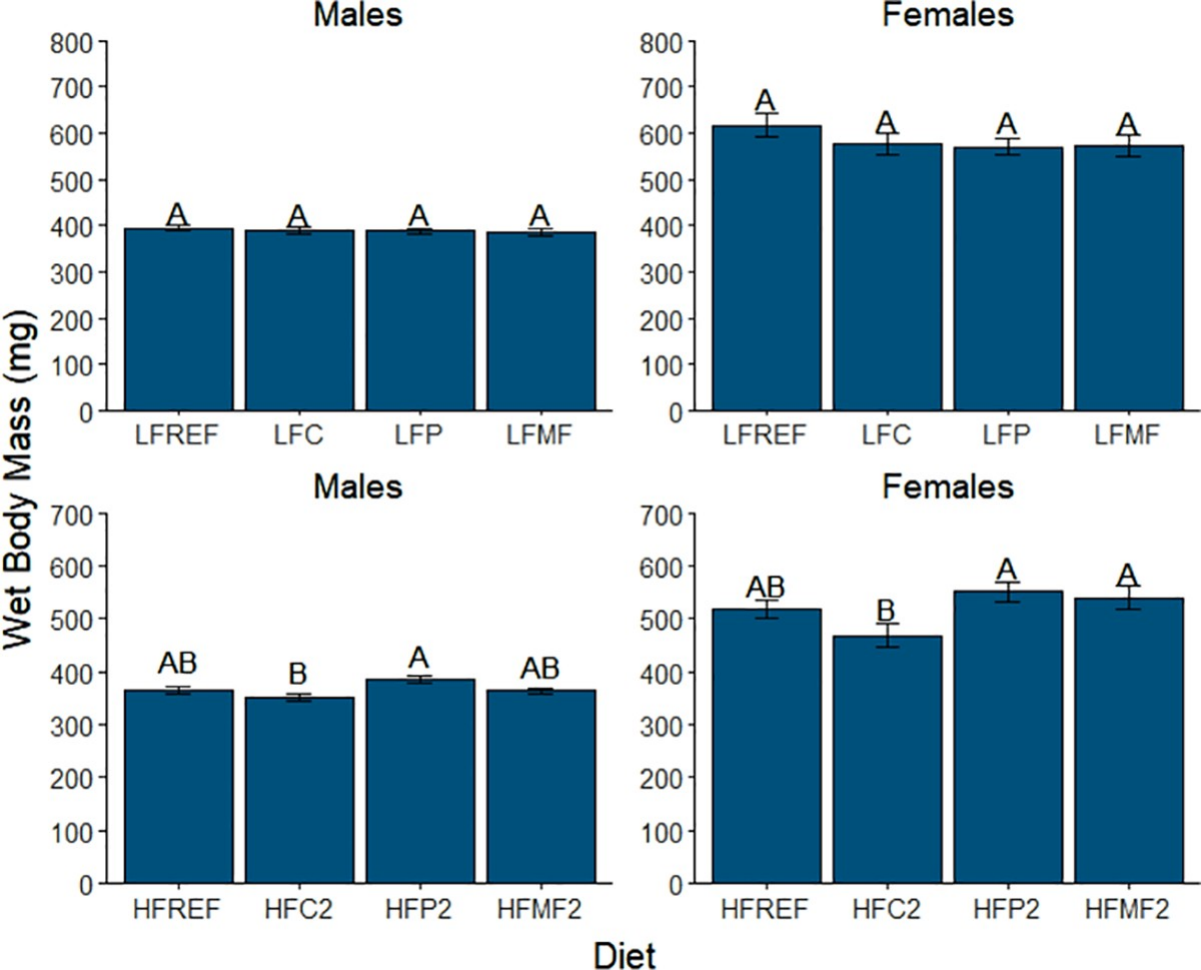
Comparison of mean wet body mass among low-fat, high-fat 2, and reference diet groups in male and female zebrafish. Error bars represent standard error of the mean. Different letters indicate between-group differences at *P*<0.05. LFREF = low-fat reference; LFC = low-fat coconut; LFP = low-fat palm; LFMF = low-fat milk fat; HFREF = high-fat reference; HFC2 = high-fat coconut 2; HFP2 = high-fat palm 2; HFMF2 = high-fat milk fat 2.

While diet and sex were observed to significantly interact in their effects on WBM, similarities between males and females were still observed for planned pairwise comparisons (Fig 2). Among low-fat diets (diet comparison group one, Table 5), significant differences in WBM were not observed in either males or females (Fig 2). Among diets in comparison group 2 (Table 5), mean WBM was significantly lower in fish fed the coconut oil high-fat 2 diet (HFC2) compared to the palm oil high-fat 2 diet (HFP2) in both sexes (males, 352 ± 6 mg vs. 385 ± 7 mg and *P* = 0.004; females, 468 ± 21 mg vs. 551 ± 35 mg and *P* = 0.001). In females, the mean WBM of fish fed the milk fat high-fat 2 diet (HFMF2, 540 ± 23 mg) was found to be significantly higher than the mean WBM for the HFC2 diet (*P* = 0.042). In contrast, a significant difference in WBM between the HFMF2 and HFC2 diets was not detected in males (*P* = 0.831).

**Fig 3:**
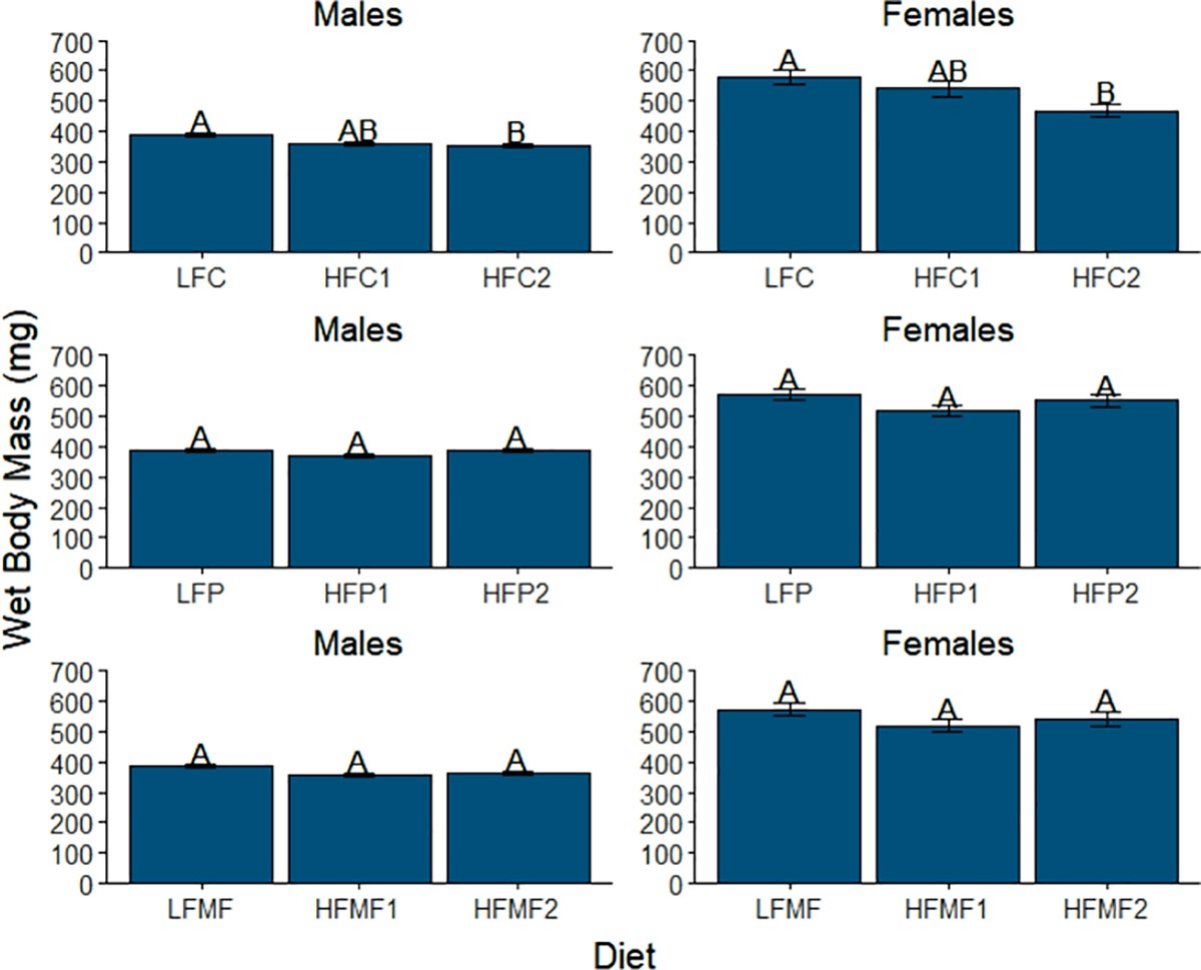
Comparison of mean wet body mass among diet groups within each saturated fat source in male and female zebrafish. Error bars represent standard error of the mean. Different letters indicate between-group differences at *P*<0.05. LFC = low-fat coconut; HFC1 = high-fat coconut 1; HFC2 = high-fat coconut 2; LFP = low-fat palm; HFP1 = high-fat palm 1; HFP2 = high-fat palm 2; LFMF = low-fat milk fat; HFMF1 = high-fat milk fat 1; HFMF2 = high-fat milk fat 2.

Males and females also exhibited similar trends in mean WBM among diets within each source of saturated fat (Fig 3). Within the coconut oil diets (comparison group three), the mean WBM of fish fed the low-fat coconut (LFC) diet (males, 394 ± 6 mg; females, 617 ± 26 mg) was observed to be significantly higher than the mean WBM of fish fed the HFC2 diet (males, *P* = 0.003; females, *P*<0.001). Mean WBM for the high-fat coconut 1 diet (HFC1) was not observed to significantly differ from the LFC or HFC2 diets. Significant differences in mean WBM were not observed within the palm oil or milk fat diets (comparison groups four and five).

### Body composition

#### Adiposity

**Fig 4:**
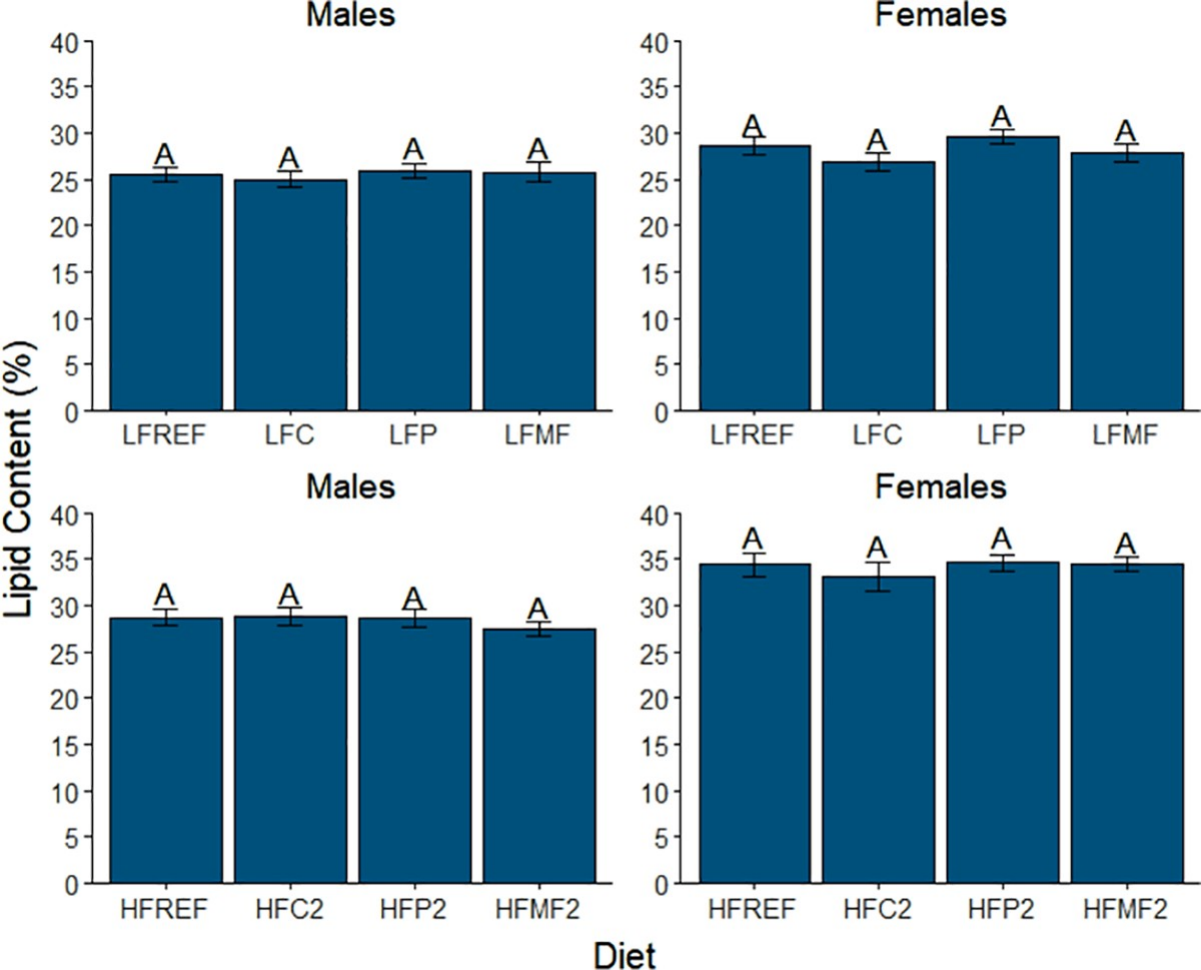
Comparison of mean lipid content (adiposity) among low-fat, high-fat 2, and reference diet groups in male and female zebrafish. Error bars represent standard error of the mean. Different letters indicate between-group differences at *P*<0.05. LFREF = low-fat reference; LFC = low-fat coconut; LFP = low-fat palm; LFMF = low-fat milk fat; HFREF = high-fat reference; HFC2 = high-fat coconut 2; HFP2 = high-fat palm 2; HFMF2 = high-fat milk fat 2.

**Fig 5:**
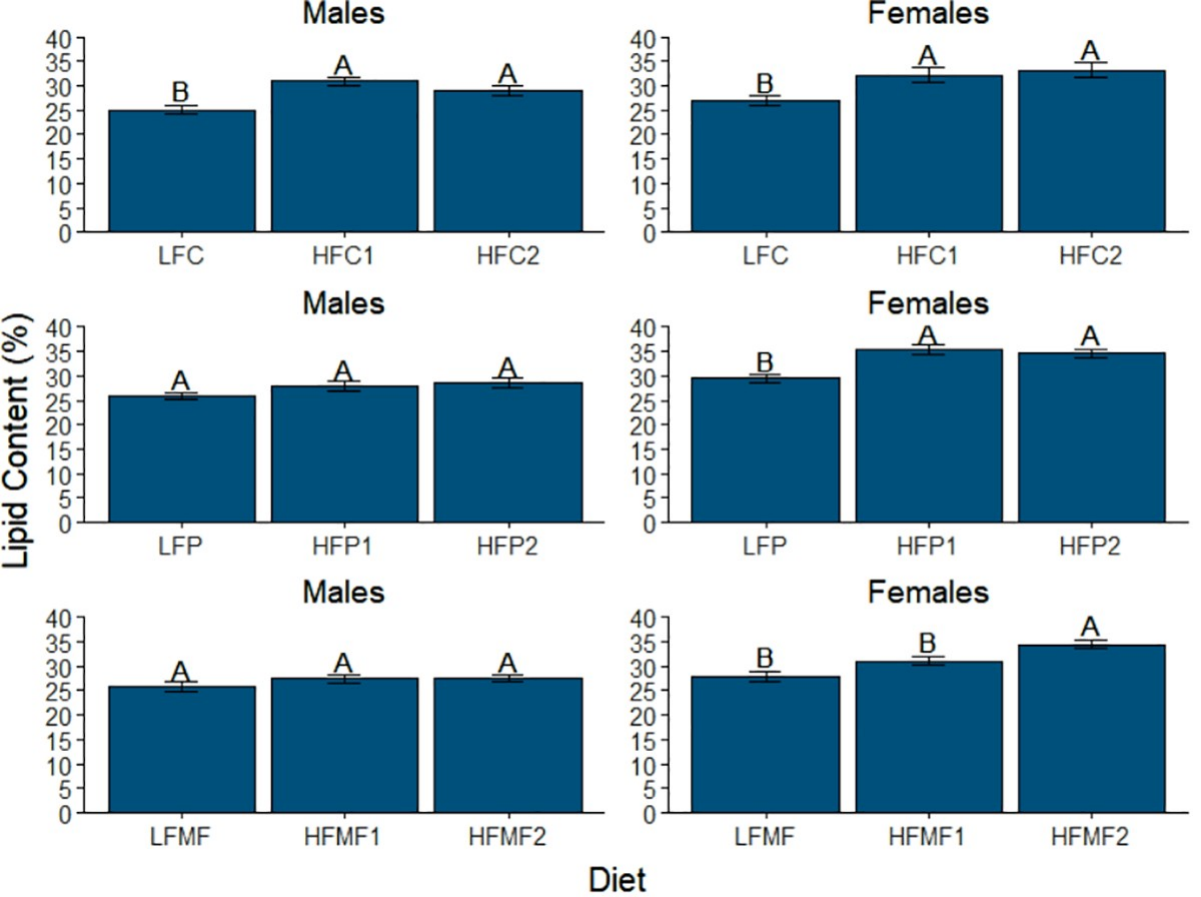
Comparison of mean lipid content (adiposity) among diet groups within each saturated fat source in male and female zebrafish. Error bars represent standard error of the mean. Different letters indicate between-group differences at *P*<0.05. LFC = low-fat coconut; HFC1 = high-fat coconut 1; HFC2 = high-fat coconut 2; LFP = low-fat palm; HFP1 = high-fat palm 1; HFP2 = high-fat palm 2; LFMF = low-fat milk fat; HFMF1 = high-fat milk fat 1; HFMF2 = high-fat milk fat 2.

In the analysis for adiposity (percent lipid content), significant main effects were observed for both diet and sex. Additionally, diet and sex interacted in their effects on adiposity (Table **7**). Within diet comparison groups one and two, no significant differences in mean percent lipid content were observed for either sex (Fig 4). For the remaining diet comparison groups, the effects of diet on adiposity varied by both sex and saturated fat source (Fig 5). Among the coconut oil diets, similar trends were observed between males and females. Mean adiposity was significantly lower in fish fed the LFC diet (males, 25.0 ± 0.9%; females, 26.9 ± 1.0%) relative to fish fed the HFC1 (males, 30.9 ± 0.7% and *P*<0.001 vs LFC; females, 32.1 ± 1.5% and *P* = 0.004 vs LFC) and HFC2 diets (males, 28.9 ± 1.0% and *P* = 0.005 vs LFC; females, 33.1 ± 1.5% and *P*<0.001 vs LFC). No significant differences were observed between the HFC1 and HFC2 diets in either sex (males, *P* = 0.215; females, *P* = 0.700).

**Table 7:** Main and interactive effects of diet, sex, and cohort on adiposity/lipid content^a^.

Model^b^	Fixed effects	Sum of squares	df	Mean squares	F-statistic	Pr(>F)
**All**	Diet	2.802	10	0.280	10.068	<0.001
	Sex	2.471	1	2.470	88.740	<0.001
	Cohort	0.076	1	0.076	2.734	0.131
	Diet*sex	0.548	10	0.055	1.971	0.035
**Males**	Diet	1.053	10	0.105	4.232	<0.001
	Cohort	0.005	1	0.005	0.212	0.645
	Diet*cohort	0.268	10	0.027	1.080	0.378
**Females**	Diet	1.912	10	0.191	6.871	<0.001
	Cohort	0.035	1	0.035	1.256	0.287
	Diet*cohort	0.241	10	0.024	0.866	0.569

^a^Log-transformed for analysis.

^b^Analyzed with mixed effects models, which controlled for tank as a random effect.

In contrast to the coconut oil diets, no significant differences in mean adiposity were observed in males among either the palm oil or milk fat diets (Fig 5). In females, trends for mean differences among the palm oil diets were similar to those observed for the coconut oil diets. Mean adiposity in females fed the LFP diet (29.5 ± 0.8%) was significantly lower than both the HFP1 diet (35.4 ± 1.0% and *P*<0.001) and HFP2 diet (34.6 ± 0.9% and *P*<0.001). No significant differences were detected between the HFP1 and HFP2 diets (*P*>0.900). In contrast, mean adiposity of females fed the high-fat milk fat 2 diet (HFMF2, 34.4 ± 0.8%) was significantly higher than females fed either the low-fat milk fat diet (LFMF, 27.8 ± 1.0% and *P*<0.001) or high-fat 1 milk fat diet (HFMF1, 31.0 ± 0.8% and *P* = 0.003). No significant differences in adiposity were observed between the LFMF and HFMF1 diets in females (*P* = 0.056).

#### Moisture content

**Fig 6:**
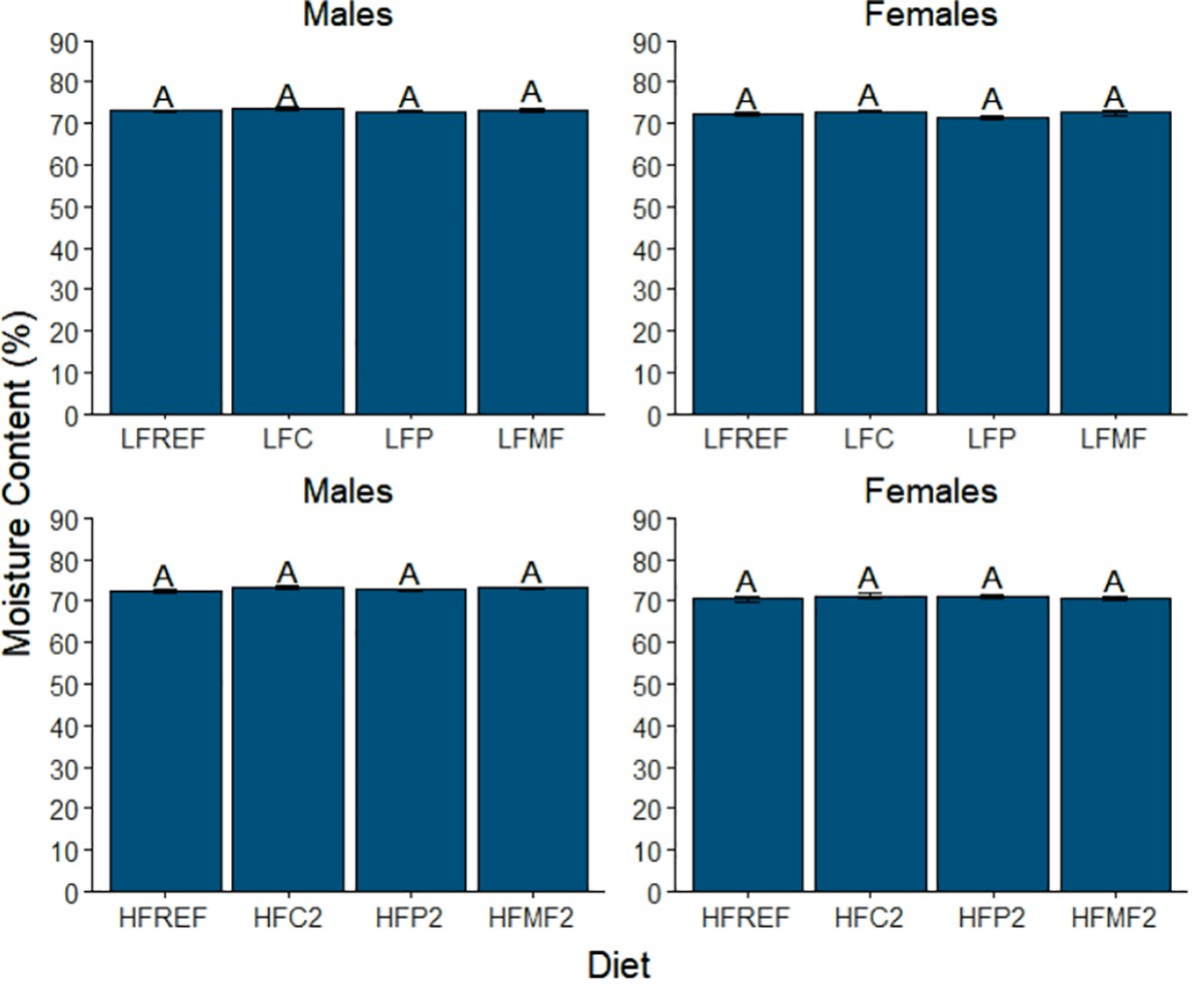
Comparison of mean carcass moisture content among low-fat, high-fat 2, and reference diet groups in male and female zebrafish. Error bars represent standard error of the mean. Different letters indicate between-group differences at *P*<0.05. LFREF = low-fat reference; LFC = low-fat coconut; LFP = low-fat palm; LFMF = low-fat milk fat; HFREF = high-fat reference; HFC2 = high-fat coconut 2; HFP2 = high-fat palm 2; HFMF2 = high-fat milk fat 2.

**Fig 7:**
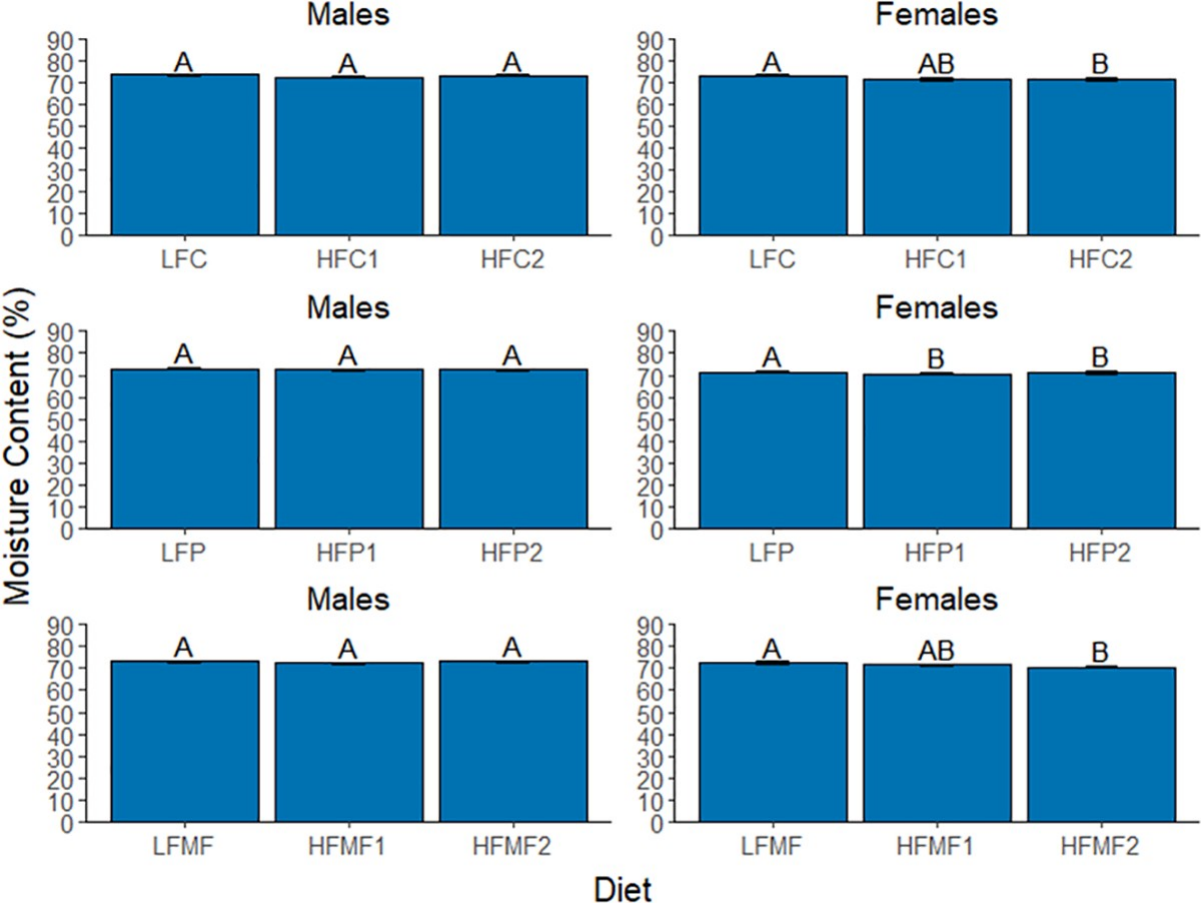
Comparison of mean carcass moisture content among diet groups within each saturated fat source in male and female zebrafish. Error bars represent standard error of the mean. Different letters indicate between-group differences at *P*<0.05. LFC = low-fat coconut; HFC1 = high-fat coconut 1; HFC2 = high-fat coconut 2; LFP = low-fat palm; HFP1 = high-fat palm 1; HFP2 = high-fat palm 2; LFMF = low-fat milk fat; HFMF1 = high-fat milk fat 1; HFMF2 = high-fat milk fat 2.

Overall, mean carcass moisture content (MC) ranged from 70.5 ± 0.5% to 73.0 ± 0.4% across diets in female zebrafish and from 72.1 ± 0.4% to 73.5 ± 0.3% in male zebrafish. Sex was not significantly associated with MC (Table 8); however, diet and sex significantly interacted in their effects on MC. In male zebrafish, diet was not a significant main effect of MC. In female zebrafish, no significant differences were detected among diets in comparison groups one and two (group one, *P* = 0.225; group two, *P* = 0.750) (Fig 6). However, MC did vary among diet groups within each source of saturated fat (Fig 7). Within the coconut oil and milk fat diet groups, MC in females fed the low-fat diets was significantly higher compared to females fed the high-fat 2 diets (coconut oil, 72.3 ± 0.4% vs. 71.2 ± 0.6% and *P* = 0.015; milk fat, 72.5 ± 0.5% vs. 70.6 ± 0.4% and *P* = 0.002). MC for the high-fat 1 diets did not significantly differ from either the low-fat diets (coconut oil, *P* = 0.167 and milk fat, *P* = 0.128) or high-fat 2 diets (coconut oil, *P* = 0.670; milk fat, *P* = 0.233). In contrast to the coconut oil and milk fat diets, MC of females fed the low-fat palm oil (LFP) diet (71.4 ± 0.5%) was significantly higher (*P*<0.001) of females fed both the high-fat 1 palm oil diet (HFP1, 70.8 ± 0.4%) and high-fat 2 palm oil diet (HFP2, 71.1 ± 3.7%). In contrast, MC did not differ between females fed the HFP1 and HFP2 diets (*P* = 0.983).

**Table 8:** Main and interactive effects of diet, sex, and cohort on moisture content^a^.

Model^b^	Fixed effects	Sum of squares	df	Mean squares	F-statistic	Pr(>F)
**All**	Diet	0.019	10	0.002	2.818	0.004
	Sex	0.042	1	0.042	6.405	0.228
	Cohort	0.009	1	0.009	13.577	0.003
	Diet*sex	0.009	10	0.001	1.315	0.044
**Males**	Diet	0.007	10	0.001	1.559	0.139
	Cohort	0.003	1	0.003	7.583	0.018
	Diet*cohort	0.004	10	0.0003	0.887	0.549
**Females**	Diet	0.017	10	0.002	1.977	0.042
	Cohort	0.008	1	0.008	9.609	0.008
	Diet*cohort	0.002	10	0.0002	0.198	0.996

^a^Log-transformed for analysis.

^b^Analyzed with mixed effects models, which controlled for tank as a random effect.

In addition to diet, cohort was also significantly associated with MC. However, while mean MC was higher in fish from cohort 2 versus cohort 1 (71.7 ± 0.1% versus 72.6 ± 0.1%, respectively), it is uncertain whether a difference of this magnitude was biologically significant.

## Discussion

Our results suggest that independent of sex, the effects of saturated fat source on body mass may be dependent on total dietary lipid intake. We report that saturated fat source, total level of dietary fat, and sex interact in their effects on body mass and adiposity in male and female zebrafish. Differences between mammals and zebrafish in the effects of coconut oil on adiposity were also revealed. Collectively, our findings indicate that in future nutrition studies, potential interactions of saturated fat with sex and level of dietary fat on metabolic health should be carefully considered.

Our results suggest that independent of sex, the different effects of saturated fat source on body mass may be dependent upon total level of dietary fat. In humans and other animals, previous evidence demonstrates that replacement of fat sources consisting primarily of long-chain triglycerides (LCTs) with sources of medium chain triglycerides (MCTs) is associated with significant weight loss [3, 6, 23, 24]. Consistent with these findings, mean body mass in male and female zebrafish fed the HFC2 diet was significantly lower than mean body mass of fish fed the HFP2 diet. Conversely, however, significant differences in body mass were not observed among the low-fat diets (comparison group 1), suggesting that with lower amounts of dietary fat, body mass is not influenced by saturated fat source. Specifically, these results suggest that replacement of other long-chain saturated fatty acid sources with coconut oil may not result in additional weight loss in conjunction with a low-fat diet.

Interactions between diet and sex on body mass also appeared to be dependent on amount of total dietary fat. While similar trends between males and females were observed among the low-fat diets, we found that for the high-fat diets, the effect of saturated source on body mass varied by sex. Sex-specific differences in the response to a high-fat diet have been observed in both humans and other animals; specifically, males have been reported to have both higher and absolute weight gain than females [25–27]. The decreased susceptibility for weight gain in females is largely attributed to the protective effects of estrogens, protecting against the detrimental effects of diet-induced obesity by increasing energy expenditure in response to higher amounts of dietary fat [9, 26]. Robison et al. (2013) showed that in zebrafish, genes associated with metabolism and oxidative stress exhibit a sex-dependent response to dietary carbohydrate manipulation [28]. Therefore, it could be hypothesized that in zebrafish, energy expenditure and metabolism associated with dietary fatty acid intake may also be sex-specific.

Among diets within each source of saturated fat, body mass only differed among the coconut oil diets, further suggesting an effect of dietary MCTs on body mass in zebrafish. However, additional studies will be required to determine the mechanisms by which coconut oil and MCT consumption affects body mass in zebrafish. We also noted that body mass for the low-fat and high-fat reference diets did not differ from their saturated low-fat or high-fat counterparts in either sex, indicating that in diets that are iso-nitrogenous and iso-caloric, replacement of dietary saturated fat with LC-PUFA sources does not impact body mass in zebrafish. This result is consistent with a previous study in rainbow trout, which found that weight gain was not altered in response to replacement of fish oil with coconut oil [29]. However, findings from similar studies in humans have been inconclusive, indicating that more research is needed in this area.

A large body of evidence has demonstrated that consumption of MCTs significantly reduces adiposity in both humans and rodents [6, 30]. In contrast to these studies, we found that inclusion of coconut oil had no effect on adiposity in zebrafish relative to other sources of saturated fat; instead, adiposity increased along with total intake of dietary fat. Similar effects of total dietary fat on adiposity were also observed in other species of fish [29, 31]. Diet-induced thermogenesis (DIT), which refers to a postprandial increase in metabolic rate, plays an important role in energy balance and has been suggested as a primary mechanism by which MCT reduces body fat [32, 33]. Given that brown adipose tissue (BAT) has been observed to positively influence DIT in humans [34], we hypothesize that differences in MCT consumption on adiposity between mammals and fish may be partially attributed to the presence (or lack of) BAT.

It was also observed that in the comparison of diets within each source of saturated fat, sex and diet interacted in their effects on adiposity. Among the three sources of saturated fat, adiposity in females differed primarily by the amount of total dietary fat, while adiposity in males was affected by the quality of dietary fat. One potential explanation for why adiposity changed in response to saturated fat source only in males could be attributed to the presence of higher amounts of visceral adipose tissue (VAT) in males. Given that visceral fat is a key risk factor for metabolic dysfunction, higher amounts of VAT could result in increased sensitivity to alterations in dietary fatty acids [26]. On the other hand, males may have a stronger drive than females to adjust feed intake relative to the energy density of the diet, which may explain why the amount of total dietary fat affected adiposity in females but not males [26].

In general, moisture content was found to be inversely associated with adiposity, which is consistent with findings from a similar study conducted in rainbow trout [29]. However, caution should be exercised in the interpretation of these results. Although statistically significant differences in moisture content were detected among diets, the variation in mean moisture content among the diets was extremely small, indicating that these differences may not be biologically significant.

Strengths of our study include the use of chemically defined diets with purified ingredients, the administration of daily rations during both the maintenance and experimental feeding periods, and statistical power to evaluate sex-specific differences for all outcomes of interest. The study design also allowed us to control for both saturated fat content and LC-PUFA content, while keeping the amounts of all other nutrients constant. Finally, the duration of the feeding trial (eight weeks) allowed us to examine long-term effects of saturated fat intake on weight gain and body composition. One major limitation of our study was the inability to measure feed intake. While we attempted to address this issue with administration of a daily ration, finding direct methods of feed intake measurement will be essential and should continue to be investigated [35].

In summary, our results demonstrate that the balance of dietary saturated fat with other components of dietary lipid consumption may be as important as the individual effects of each type of saturated fat. Dietary fat quantity interacted with saturated fat source on weight gain and adiposity. Also observed was the lack of an effect of dietary replacement of saturated LCT sources with coconut oil on adiposity in zebrafish. However, our observations for both adiposity and body mass indicated that the partitioning and utilization of dietary lipid was sex-specific. Findings from our study not only emphasize the need to re-assess recommendations for dietary fat consumption, they also further validate the zebrafish model system for examining sex-specific effects of dietary lipid consumption on obesity.

## Supporting information

S1 Data Terminal data for body mass and body composition. (XLSX)
